# Clinical factors associated with fatigue over time in paediatric oncology patients receiving chemotherapy

**DOI:** 10.1038/sj.bjc.6604434

**Published:** 2008-06-24

**Authors:** C-H Yeh, Y-C Chiang, L Lin, C-P Yang, L-C Chien, M A Weaver, H-L Chuang

**Affiliations:** 1 Graduate Institute of Nursing Science, Chang Gung University, Kwei-San, Tao-Yuen, Taiwan; 2 Department of Nursing, Chang Gung Institute of Technology, Kwei-San, Tao-Yuen, Taiwan; 3 School of Nursing, University of North Carolina at Chapel Hill, Chapel Hill, NC, USA; 4 Department of Hematology and Oncology, Chang Gung Children's Hospital, Kewi-San, Tao-Yuen, Taiwan; 5 Department of Biostatistics, University of North Carolina at Chapel Hill, Chapel Hill, NC, USA; 6 Family Health International; 7 Ten-Chen General Hospital, Taiwan

**Keywords:** fatigue, paediatric oncology patient, clinical factors, chemotherapy, corticosteroids

## Abstract

The purpose of this study was to investigate the relationships between clinical factors (including haemoglobin value, chemotherapeutic agents, and corticosteroid use) and changing patterns of fatigue before and for the next 10 days following the start of a new round of chemotherapy in children with cancer. A prospective longitudinal design was used to collect data from 48 paediatric oncology patients who were about to begin a new round of chemotherapy and their parents. Fatigue levels were assessed using multidomain questionnaires with three categories of patient self-report (including ‘General Fatigue’, ‘Sleep/Rest Fatigue’, and ‘Cognitive Fatigue’) and four categories of parent proxy-report (including ‘Lack of Energy’, ‘Unable to Function’, ‘Altered Sleep’, and ‘Altered Mood’). The findings suggest that fatigue from both patient self-report and parent proxy-report changed significantly over time. The major findings from this study are that patients have more problems with fatigue in the first few days after the start of a cycle of chemotherapy. Corticosteroid use and haemoglobin value were associated with significant increases in fatigue that were sustained for several days and reached the highest level of fatigue at day 5 for those receiving concurrent steroids. The association of chemotherapeutic agents with fatigue varied between patient self-report and parent report, but the type of chemotherapeutic agents used was not associated with most changes in fatigue.

Fatigue is one of the most frequent and severe symptoms experienced by paediatric oncology patients during treatment (Hockenberry-Eaton *et al*, 1998; Hinds *et al*, 1999; Wolfe *et al*, 2000; Collins *et al*, 2002). It is also among the most distressing symptoms reported in the terminal phase of their illness (Wolfe *et al*, 2000; Jalmsell *et al*, 2006; Theunissen *et al*, 2007). However, very few quantitative studies have reported on fatigue in paediatric oncology patients (Varni *et al*, 2002; Langeveld *et al*, 2003). Aggressive treatment of paediatric oncology patients is focused on cure; therefore, side effects, such as fatigue, that result from treatments may be ignored by clinicians (Hockenberry-Eaton and Hinds, 2000) or considered as unavoidable symptoms that need to be endured (Gibson *et al*, 2005).

The development of the proposed models for fatigue is based on adult oncology patients (Piper, 1986; Nail and Winningham, 1993; Wessely *et al*, 1998). The management of fatigue is an important clinical issue because fatigue is recognised as having a greater impact on patients than other mental or physical consequences of cancer or its treatment (Stone *et al*, 2000; Ahlberg *et al*, 2005). Fatigue is complex and is often associated with multiple physiological and emotional sequelae. Few studies have examined the aetiology of fatigue associated with chemotherapy treatment (CTX). In a study that evaluated the epidemiology of cancer-related fatigue in 419 adult cancer patients, 74% of the patients considered fatigue as a symptom to be endured and 80% of the oncologists believed fatigue is undertreated (Vogelzang *et al*, 1997). Fatigue may exist before, during, and after CTX for cancer (Langeveld *et al*, 2000). Stasi *et al* (2003) proposed that fatigue would be more intense in the first few days after CTX and then would gradually abate until the next course of CTX. However, little empirical evidence exists to support this statement.

Factors that may contribute to fatigue intensity, such as anaemia (Wang *et al*, 2002) and the role of cortisol (Payne, 2004), are not well documented. Corticosteroids are essential in the treatment of acute lymphoblastic leukaemia, which represents the majority of cancers diagnosed in childhood (Gaynon and Lustig, 1995). However, the relationship between fatigue and treatment protocols including the use of corticosteroids has not been investigated in detail.

Identifying the specific factors associated with the fatigue that accompanies CTX could provide important information to clinicians and researchers. Clinicians would be able to assess and treat these symptoms, not only making the children more comfortable but increasing patient and parent satisfaction as well (Braud *et al*, 2003). It is important for clinicians to acknowledge the impact of fatigue and, whenever possible, provide therapeutic interventions to decrease it. Thus, the purpose of this study was to investigate changing patterns of fatigue by enrolling paediatric oncology patients just before the start of a new round of CTX and then following them for 10 days. Factors that may contribute to fatigue, including chemotherapeutic agents, corticosteroid use during the study period, and haemoglobin value, were also examined.

## Materials and methods

A prospective longitudinal design was used to evaluate fatigue in paediatric oncology patients who received CTX. Each subject and their parent provided data about the child's fatigue experiences before CTX and each of the next 10 days.

### Participants

Participants were children with cancer and their parents. Children were eligible for this study if they were aged 7–18 years, were diagnosed with cancer, were prescribed CTX, and were not receiving concomitant radiation or had not undergone bone marrow transplantation. All children were prescribed one of the following chemotherapeutic agents: methotrexate, vincristine, etoposide, cisplatin, cytarabine, epirubicin, or cyclophosphamide. Data of concomitant corticosteroids used as part of the protocol for the CTX cycle were also collected. If a patient received corticosteroids for treatment purposes, other than a component of their CTX protocol, they were excluded from this study. Corticosteroids used included prednisolone, hydrocortisone, or dexamethasone. Owing to different CTX protocols, participants were enrolled in this study when they were about to receive a new round of CTX and their first fatigue assessment could be made on the day before the new round was started. Additional assessments were made 24 h after the CTX had begun (i.e., day 02) and then daily for the next 9 days. The research team selected 11 days as the timeframe to allow sufficient time for symptoms to change, as well as for administration of chemotherapeutic agents and steroids. In most CTX protocols, these agents were administered within a week, and this allowed at least 3 days to collect data on the resulting symptoms. Fifty-two patients were approached and two patients declined because of parental time constraints. Two other patients who had been on CTX for a more extended period of time (greater than 30 months) were also excluded. In total, data were collected from 48 patients and 48 parents. Table 1 presents the detailed demographic and diagnostic characteristics of the children.

### Measures

The conceptual definition used in this study was defined by our previous qualitative study of exploring the fatigue experiences reported by Taiwanese children with cancer (Chiang *et al*, 2008b). Fatigue is defined as ‘a phenomenon that includes both physical and psychosocial sensations (e.g., tiredness, lack of energy, and lack of motivation) as well as consequences (e.g., lying on the bed and being unable to engage in physical activities)’ (Chiang *et al*, 2008b). Owing to the limited number of fatigue measurement tools available at data collection, two existing instruments were used to collect data for this study. The PedQL Multidimensional Fatigue was administered to the patients and the Parent Fatigue Scale (PFS) was used for the parents. Both were originally designed and tested in English. For our studies, both measures were translated from English into Chinese language. The detailed process of translation and back translation is described in another publication (Chiang *et al*, 2008a).

### PedsQL Multidimensional Fatigue scale

The PedsQL Multidimensional Fatigue scale (Varni *et al*, 2002) was used to measure patients' self-reported fatigue symptoms. It consists of three subscales, including ‘General Fatigue’ (six items), ‘Sleep/Rest Fatigue’ (six items), and ‘Cognitive Fatigue’ (six items). All items used a five-point Likert response set, ranging from 1=‘not at all’ to 5=‘always’. To assess the detailed pattern of fatigue during chemotherapy for paediatric oncology patients, a new version of the PedsQL Multidimensional Fatigue scale, one that was intended for daily assessment of fatigue for the past 24 h, was developed and tested (Chiang *et al*, 2008a). The reliabilities of the three subscales and total scale in the 24-h version ranged between 0.83 and 0.93 (Cronbach's *α* coefficient) and the validity was also evident with high correlation with the Chinese version of the Fatigue Scale-Children (Pearson correlation values ranged from 0.36 to 0.56) (Chiang *et al*, 2008a). In the original instrument, higher scores indicated fewer symptoms of fatigue. To compare the fatigue change patterns for patients and parents, the PedsQL Multidimensional Fatigue Scale used in the current study was rescored so that higher scores indicated that patients had increased fatigue. Cronbach's *α* values for the various subscales ranged from 0.91 to 0.95 for this study.

### The Parent Fatigue Scale

The PFS (Hockenberry *et al*, 2003) was used to measure parental perception of their child's fatigue over the previous 24 h. The PFS included four fatigue subscales: ‘Lack of Energy’ (six items), ‘Unable to Function’ (three items), ‘Altered Sleep’ (four items), and ‘Altered Mood’ (four items). All items used a five-point Likert response set, ranging from 1=‘not at all’ to 5=‘always’. Higher scores indicated that parents perceived that their child experienced greater fatigue. Good reliability has been reported in previous studies with this instrument (Cronbach's *α* for the total scale was 0.88) (Hockenberry *et al*, 2003). Strong correlation with the child's self-report of fatigue, as well as convergent validity with other well-established measures, was also reported (Hockenberry *et al*, 2003). Cronbach's *α* values for the subscales ranged from 0.61 to 0.87 for this study.

### Demographic information

Demographic and relevant data were collected from medical records and included age, gender, cancer diagnosis, haemoglobin level, body weight, and treatment information. Haemoglobin level at the baseline assessment (i.e., from blood drawn on the day before the first dose of that round of CTX) was available for all patients. For some patients (*n*=17), haemoglobin was assessed only at baseline. However, the remaining patients (*n*=31) had at least one haemoglobin assessment during follow-up. Use of chemotherapeutic agents and corticosteroids before baseline assessment and on each day during data collection was also recorded.

### Procedures

The study began after receiving approval from the Human Subjects Review Committee at Chang Gung Children's Hospital in Taiwan. The hospital's established procedures for protecting confidentiality were strictly followed. The patients in the study setting who were about to receive CTX at the hospital ward and who met the sampling criteria were approached by a trained research assistant (RA). The parents received verbal and written explanations of the study and procedures and were asked about their willingness to participate in this study along with their sick child. After parental consent and child assent were obtained, they were scheduled and interviewed in person at the hospital wards by a trained interviewer. Each patient and their parent were interviewed independently by face-to-face interviews in the hospital or by telephone if the patient was no longer hospitalised at the time of data collection. The RA administered the questionnaire package in person at the hospital for the baseline assessment and collected follow-up assessment data either in person or by phone if a patient had been discharged during the data collection period. Following data collection, identity of patients and parents was removed and all identifiable materials were stored in a locked office.

Data were collected at 11 time points, baseline (pre-CTX) and daily for 10 consecutive days. The data were collected at the same time each day (i.e., in the morning before the CTX began for that day).

### Data analysis

Descriptive statistics were calculated for the demographic characteristics of the patients. It was expected that the items within each subscale of fatigue could contribute differently to the underlying fatigue construct. Thus, rather than simply adding the scores for all items within a subscale, the score for each subscale was calculated using the factor scores obtained from a single-factor analysis. The ranges of scores for each subscale were converted to the range of 1–5 so that the different fatigue levels could be directly compared. A multivariate mixed-effects model (using the MIXED Procedure in SAS) (SAS Institute Inc., 2006) was used to capture the longitudinal outcome measures (modelling covariance across time) as well as the high correlation between the multidomain outcomes (modelling covariance across the multivariate observations, such as General, Sleep/Rest, and Cognitive Fatigue for patient-reported data) (Galecki, 1994).

To account for the multivariate nature of the data (multiple correlated symptoms over time), a direct-product covariance structure (UN@AR(1)), available in PROC MIXED (SAS Institute Inc., 2006), was used. Patient self-reported and parent proxy-reported data were modelled separately, because the instruments used measured slightly different concepts associated with fatigue. The model included fixed effects for time, cumulative CTX agent and cumulative corticosteroid use before baseline assessment and through each study day, and current haemoglobin level. Missing current haemoglobin values were imputed using the most recently observed haemoglobin values for each patient (for 17 patients, baseline haemoglobin value was used throughout). For each study day, chemotherapeutic agent use and corticosteroid use were each coded as 0=‘not used’ and 1=‘used’. Then, to examine the cumulative relationship with fatigue factors, the cumulative use of chemotherapeutic agents and corticosteroids before the baseline assessment and through each day in this cycle of CTX was calculated and used as a covariate in the model. Model effects were tested at a significance level of 0.05. Data analyses were performed using SAS software, version 9.1.3 (SAS Institute Inc., 2006).

## Results

### Characteristics of participants

The patients included 26 males and 22 females. The mean age of the patients was 11.51 years (range 7–17, s.d.=2.95 years). Cancer diagnoses included leukaemia (*n*=25), lymphoma (*n*=2), solid tumour (*n*=17), and brain tumours (*n*=4). The average duration for receiving CTX before the current cycle was 4.37 months (range 0–21.23, s.d.=4.53 months), including eight patients who were newly diagnosed and were receiving their first round of CTX; 10 had been treated for less than 2 months, 11 between 2 and 4 months, and 19 had been treated for more than 4 months. Twenty-six (54%) patients received corticosteroids. The parents included 41 mothers, 6 fathers, and 1 adult family member.

### Change patterns of fatigue

**Figure 1:**
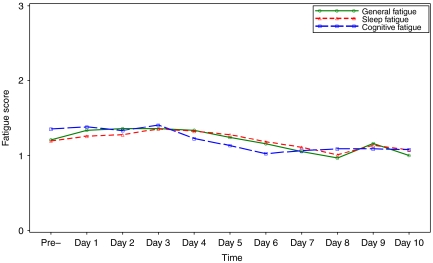
Unadjusted factor mean scores of fatigue change patterns over time reported by children.

**Figure 2:**
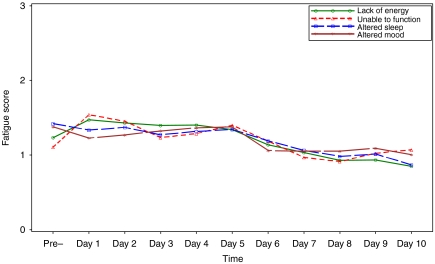
Unadjusted factor mean scores of fatigue change patterns over time reported by parents.

Figures 1 and 2 present the unadjusted means of factor scores of fatigue for patients' self-reports and parents' proxy-reports, respectively. Patterns of change in fatigue were determined across the 11 time points. After adjusted by cumulative days of CTX, steroids used before baseline assessment, haemoglobin value, and cumulative chemotherapeutic agent and corticosteroid use for each study day, time was associated with changes in the fatigue scores (*P*<0.001) for patient self-reported data, which indicates that patients in this study report statistically significant fatigue when the subscales of fatigue were treated as a multidomain construct. General Fatigue increased after CTX was administered to its highest level on day 2 and then gradually decreased over time. Sleep/Rest Fatigue levels also increased after CTX and reached the highest level on day 3, but this change was not significant over 11 time points (*P*=0.21) after controlled by other factors. Cognitive Fatigue did not change significantly over the course of the study (*P*=0.95), although its observed change pattern appears similar to subscales of General Fatigue and Sleep/Rest Fatigue.

For parents' proxy-reported fatigue scores, all four of the domains of fatigue changed significantly over time (*P*<0.001). From Figure 2, the unadjusted fatigue mean scores of Lack of Energy and Unable to Function increased immediately after CTX was administered and reached the highest levels of fatigue on day 1 and then gradually decreased until day 10; however, Unable to Function had a small peak at day 5. Altered Mood reached its highest intensity level on day 5 and then decreased thereafter. Altered Sleep showed a small fluctuation until day 5, but generally decreased until day 10.

### Factors associated with the patterns of change in fatigue

For patients' self-reported fatigue scores, the cumulative use of corticosteroids before baseline assessment and during study day, chemo drugs before baseline assessment, and haemoglobin were significantly associated with increased fatigue for all three subscales (i.e., General Fatigue, Sleep/Rest Fatigue, and Cognitive Fatigue). Chemotherapeutic drug administration during the study days was not associated with fatigue change.

**Figure 3:**
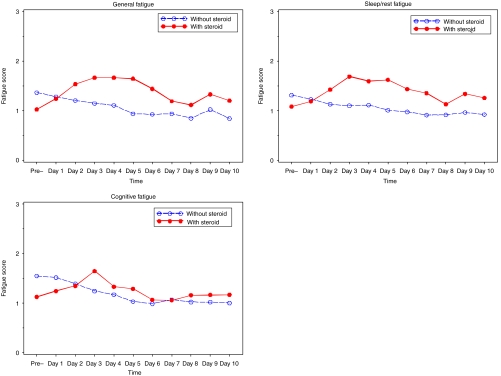
Unadjusted mean scores of fatigue change pattern for patients with and without corticosteroid use over time: patient self-report.

Figure 3 compares the unadjusted mean scores of fatigue change patterns for patient self-reported data over time for those patients who ever received corticosteroids and those who did not (descriptive data for fatigue factor scores are not presented here but are available on request). Among those patients who did not receive corticosteroids, the General Fatigue, Sleep/Rest Fatigue, and Cognitive Fatigue scales decreased fairly consistently. However, for those patients who were given at least 1 day of corticosteroids during this period, all three scales increased from baseline to day 3 or 4. Day 4 or 5 had the maximum differences on the Sleep/Rest Fatigue scale. After controlling for haemoglobin and cumulative use of CTX agents, the means of all three scales were significantly higher for those using corticosteroids consecutively than for those who did not.

**Figure 4:**
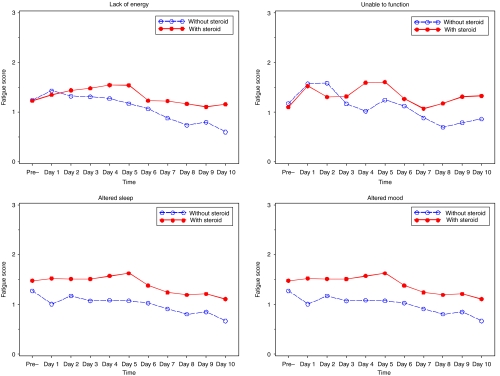
Unadjusted mean scores of fatigue change pattern for patients with and without corticosteroid use over time: parent proxy-report.

For parent proxy-reported data, higher levels of Lack of Energy, Altered Sleep, and Altered Mood were significantly associated with increasing cumulative corticosteroid use and a decrease in haemoglobin value (*P*<0.001). Figure 4 shows that the unadjusted fatigue mean scores of corticosteroids appear to increase all fatigue scores in the beginning, even though the increase is very slow, besides Unable to Function, which had a sudden jump at day 1, but decreased later, and had another jump at Day 5. In addition, before Day 2, patients who did not use corticosteroids had a higher fatigue score in Unable to Function than those without corticosteroid use. Generally speaking, all of the unadjusted fatigue mean scores reached their highest average value on day 4 or 5 for patients who received corticosteroids. After controlling for haemoglobin and cumulative use of CTX agents and corticosteroids, the mean of the Lack of Energy, Altered Sleep, and Altered Mood scores was significantly higher for those patients who used corticosteroids than for those who did not; although nonsignificant, a consistent pattern was observed for the Unable to Function scale as well.

## Discussion

This prospective longitudinal study is the first to document clinical factors associated with patterns of change in fatigue during a round of CTX, experienced by children with cancer in Taiwan. The major findings from this study are that patients have more problems with fatigue in the first few days after the start of a cycle of CTX. The administration of corticosteroids and haemoglobin value are associated with significant increases in fatigue that are sustained for several days and reach the highest level of fatigue at Day 5 for those using steroids consecutively. Of note, parent proxy-report data and patient self-report data indicated different factors associated with changes in fatigue. We preface the detailed discussion of our results by listing several limitations that should be considered. First, although all children suffered from cancer and all had been receiving CTX for less than 22 months, several different CTX protocols were being used to treat the children in this study. Additionally, there were several different cancer diagnoses among the children studied. As an example, children with brain tumours may have had more pre-existing issues with fatigue than some of the other children. Lastly, all children had haemoglobin levels drawn on day 1, before starting their round of CTX; however, 35% of the children did not have subsequent haemoglobin levels drawn during the next 10 days of data collection. Another limitation of this study that may affect its interpretation is that the assessment of fatigue by patient self-report and that by parent proxy-report are similar but showed slight variation. Thus, it is not possible to directly examine the validity of proxy-reported fatigue for patient self-reported and parent proxy-reported data. The reason for choosing these two versions is the limited established validity of the instruments in the Taiwanese population. Several authors agree that fatigue is a subjective construct (Hinds *et al*, 1999; National Comprehensive Cancer Network, 2006; Ream *et al*, 2006) and that it should be assessed directly by the patients self-report. However, the fact that children who are ill are providing these data and their cognitive and linguistic levels vary makes using only patient self-reported data less reliable (Chang and Yeh, 2005). Furthermore, brief questionnaires are needed to minimise the burden to young subjects. For example, in a study comparing quality of life measured using both child self-report and parent proxy-report (Chang and Yeh, 2005), it was determined that children younger than 12 years of age provided different perspectives of subjective domains of quality of life from parental report. In addition, when parents assess patient's fatigue, parents' own fatigue may influence their judgment of patient's fatigue.

The widely recognised definition of fatigue in paediatric oncology patients is ‘profound sense of being physically tired, or having difficulty with body movement such as using their arms and legs, or opening their eyes’ for children (Hockenberry *et al*, 2003, p 320) and ‘a changing state of exhaustion that could include physical, mental and emotional tiredness’ for adolescents (Hockenberry *et al*, 2003, p 320). The behaviours of fatigue are observable by parents and clinicians. Thus, the assessment of fatigue for paediatric oncology patients needs to take into account parent proxy-report. Parents know their children better than anyone else, and should be listened to when stating concerns about their child's fatigue. There is no ‘gold standard’ for fatigue assessment in children and there are few studies, even in English, that report on the validity of children's self-report data on fatigue. Thus, we collected patient self-reported and parent proxy-reported data simultaneously, to provide additional data on fatigue, particularly the data collected from younger children.

This study is the first to document the relationship between corticosteroids and fatigue. Corticosteroid use was associated with fatigue in most domains reported by either patients or parents. Most of the literature pertaining to adults have documented the impact of chemotherapeutic agent use on fatigue, but there was no literature to explain the relationship between corticosteroid use and fatigue in children receiving chemotherapy.

Corticosteroid use was associated with higher fatigue scores and reached the highest at day 5. The mechanism for the corticosteroid-related fatigue found in this study is not clear. Increased fatigue levels associated with the use of corticosteroids are in conflict with the theory of hypothalamic-pituitary-adrenal axis in which fatigue is assumed to be related to low circulating levels of cortisol (Jones *et al*, 1998; Payne, 2004). Patients in this study received long term and high doses of corticosteroids, which may lead to complications of corticosteroid use, such as sleep disruption, and therefore increasing the possibility of fatigue. Further study is needed to examine other possible factors that may cause fatigue, such as different cancer diagnoses, CTX agents, and pre-existing sleep problems.

The patients included in this study received a variety of CTX protocols, combining different chemotherapeutic agents, with or without corticosteroids. Owing to the sample size of this study, we did not analyse the relationship between fatigue levels and the use of corticosteroids with specific chemotherapeutic agents. The underlying mechanism of fatigue is not yet clear (Cleeland *et al*, 2003); this makes it difficult to develop scientific foundations for intervention to relieve fatigue.

As the sample size used is small, generalising these results without further study would be premature. However, this study can be used by clinicians and researchers as a stimulus to conduct more clinical assessments and conduct replications and expansions of the present study. Clinicians can be more vigilant about assessing fatigue and noting changes in the child's fatigue level as it relates to length of time since CTX and steroids were administered. They could also note how fatigue relates to any nausea, vomiting, or pain as well as changes in laboratory values. Clinicians could also record the parent's perception of their child's fatigue. Researchers could expand this study by recruiting more children who had the same diagnosis and CTX protocols. To conduct such studies it might require collecting data from different places.

Our study supports the assumption that lower haemoglobin level is a significant contributor to fatigue as previously described in the literature (Shafqat *et al*, 2005). Routine clinical laboratory check for haemoglobin in the current study setting varies, ranging from 3 days to 1 month, depending on a patient's treatment protocol. The study findings do indicate that fatigue is likely to vary over the course of time and treatment and that the timing of haemoglobin measurements may not be frequent enough to catch the rapidly changing fatigue level. Although the literature does not report a conclusive relationship between haemoglobin and fatigue (Hockenberry *et al*, 2003), this finding does suggest that frequent haemoglobin check might increase the clinicians' understanding of fatigue that accompanies CTX.

## Figures and Tables

**Table 1:** Demographic characteristics of patients

**Variables**	***n***	**%**
*Age*		
Mean (s.d.) (range)	11.51 (2.95) (7–17)	
		
*Gender*		
Male	26	54
Female	22	46
		
*Duration since initial diagnosis (months)*		
Mean (s.d.) (range)	4.37 (4.53) (0–21)	
		
*Type of disease*		
Leukaemia	25	52
Lymphoma	2	4
Solid tumour	17	36
Brain tumour	4	8
		
*Illness stages*		
Newly diagnosed within 2 months	18	38
Remission on treatment	30	62
